# Block sequential adriamycin CMF – optimal non-myeloablative chemotherapy for high risk adjuvant breast cancer?

**DOI:** 10.1038/sj.bjc.6600660

**Published:** 2002-12-02

**Authors:** D A Cameron, A Anderson, E Toy, T R J Evans, J H Le Vay, I C S Kennedy, R J Grieve, T J Perren, A Jones, J Mansi, J Crown, R C F Leonard

**Affiliations:** Department of Oncology, Western General Hospital, Edinburgh, UK; Department of Oncology, Velindre Hospital, Cardiff, UK; Department of Medical Oncology, University of Glasgow, Beatson Oncology Centre, Western Infirmary, Glasgow, UK; Suffolk Oncology Centre, Ipswich, UK; Waikato Hospital, Hamilton, New Zealand; Department of Radiotherapy, Walsgrave Hospital, Coventry, UK; CRUK Clinical Centre in Leeds, St. James's University Hospital, Leeds Teaching Hospitals NHS Trust, Leeds, UK; Department of Oncology, Royal Free Hospital, London NW3 2QG, UK; St. George's Hospital Medical School, Cranmer Terrace, London SW17 0RE, UK; St. Vincent's Hospital, Dublin, Ireland; Department of Oncology, Singleton Hospital, Swansea, UK

**Keywords:** anthracycline, adjuvant, breast cancer, survival

## Abstract

After the publication of the 10-year survival data from Milan on the adjuvant use of the block sequential regimen consisting of four cycles of adriamycin followed by eight cycles of intravenous CMF, many centres adopted this as standard of care for high risk, multiple node-positive breast cancer. For this reason it was identified as the standard arm for the Anglo-Celtic adjuvant high-dose chemotherapy trial. This study reports on the experience of this regimen in 329 women with early breast cancer involving at least four axillary nodes, who were treated outside any adjuvant chemotherapy trial. At a median follow-up of 3 years, the overall 5-year disease-free survival is 61%, and the overall survival is 70%. These data confirm the efficacy of this regimen in non-trial patients, and, for the same high risk subgroup, indicate that this approach offers an outcome at least as good as that seen in the CALGB 9344 AC-Taxol arm, and the NCIC days 1 and 8 CEF.

*British Journal of Cancer* (2002) **87**, 1365–1369. doi:10.1038/sj.bjc.6600660 www.bjcancer.com

© 2002 Cancer Research UK

## 

The place of anthracyclines in the adjuvant treatment of early breast cancer has been established by a number of individual trials in addition to recent overviews (Early Breast Cancer Trialists Collaborative Group, 1998; 2002). However, the optimal anthracycline regimen remains less well defined and opinions vary as to whether women with early breast cancer gain most from several identical cycles of chemotherapy, or sequential ‘blocks’ of different agents or combinations of agents.

In the early 1980s, as a consequence of the theoretical modelling of the Goldie–Coldman hypothesis (Goldie and Coldman, 1979; Goldie *et al*, 1982), several clinical trials were established to determine if alternating cycles of chemotherapy would be better at eradicating sub-clones of resistant malignant cells than the sequence of several cycles of one regimen followed by several of another, non-cross-resistant combination. Gianni Bonadonna and co-workers initiated one such study in Milan for women with multiple node-positive breast cancer. The first report from that study concluded that not only was the theoretically better alternating regimen not superior, but it was quite clearly inferior to the block sequential approach (Buzzoni *et al*, 1991). A subsequent theoretical analysis sought to explain this result (Norton and Day, 1992), and its validity was confirmed with the publication of the 10-year outcome data (Bonadonna *et al*, 1995). However, irrespective of the explanation, the success of this regimen in this high risk group led it to be adopted in many European and North American centres as standard of care for women with four or more involved axillary nodes. It was also adopted as the control arm of the Anglo-Celtic adjuvant high-dose chemotherapy trial, expected to report for the first time in 2002.

More recently, two other adjuvant breast cancer trials have demonstrated a clear advantage of a newer regimen over an older one. Firstly, the NCIC MA5 trial in which methotrexate was replaced by epirubicin (Levine *et al*, 1995), and more recently, the CALGB 9344 study, which although reported in abstract form only to date, suggests that the addition of a block of four doses of single agent taxol may improve overall survival (Henderson *et al*, 1998).

This study is therefore an audit of the experience of the adriamycin-CMF regimen in non-trial patients from several breast cancer units, all participants in the Anglo-Celtic trial, in order to determine how it compares to these two other pivotal adjuvant regimes.

## METHODS

### Patients

Ten centres participating in the Anglo-Celtic trial (eight in the UK, one each in Ireland and New Zealand) agreed collectively to audit the outcomes of those patients not enrolled into that trial, for reasons of patient or clinician choice, but treated nonetheless with the control arm used in the trial, the adriamycin-CMF regime. All patients had to have had potentially curative loco-regional surgery for their first diagnosis of invasive breast cancer, and to have been staged according to local practice as having breast cancer stages II–IIIA only. Patients with locally advanced tumours were not included. For the purposes of this audit, there were no exclusions on the basis of haematological or biochemical parameters, or on the basis of other medical diagnoses. Patients included had been treated with the adriamycin-CMF sequential regimen, having been offered that by their treating physician on the basis of his/her own assessment of their fitness for this regimen.

The data were then supplied in an anonymised fashion to the Edinburgh Breast Unit on request, and had therefore not been subjected to any external data audit. The data were then combined, and no analyses of the results in the individual centres have been performed.

### Statistics

Data were collected from the various centres and combined. Patients treated with this same regimen with less than four confirmed involved axillary nodes were not included. Disease-free and overall survival data were calculated from date of first chemotherapy administration, and analysed using the Kaplan–Meier method. Data on dose reductions and/or dose delays were provided by the centres, but were missing for just over one third of the patients. Analyses on dose delivery have therefore been confined to the 64% of patients for whom data were available. No data on the actual number of days' delay were available, and so true dose intensity cannot be calculated. It has been estimated using the percentage of planned dose actually delivered for the majority of patients for whom data were available:


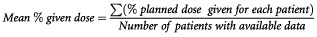


### Treatment

This consisted of four cycles of single agent doxorubicin (adriamycin) 75 mg m^−2^, given as an i.v. bolus once every 3 weeks, followed by eight cycles of cyclophosphamide 600 mg m^−2^, methotrexate 40 mg m^−2^ and 5-fluorouracil 600 mg m^−2^, all given as i.v. bolus once every 3 weeks. Decisions about dose modification and/or treatment delay were left to the discretion of the local practice. Radiotherapy was given as per local practice. All women with Oestrogen (ER) and/or Progesterone (PgR) positive breast cancer were to receive tamoxifen (or equivalent) for 5 years.

## RESULTS

### Patients

A total of 329 patients were identified who had been treated with the adriamycin-CMF regimen outside a clinical trial, and known to have at least four involved ipsilateral axillary nodes. The median number of involved nodes was eight, with a range of 4–36. The median age of the patients was 49 years, but with a wide range from 26 to 73 years.

### Tumours

Median size of the tumours was 28 mm, ranging from 6 to 200 mm. Grade was assessed by the modified Bloom Richardson method, and results were available in 300 women, of whom 167 (56%) had grade 3 tumours. One patient had bilateral cancers, and there were almost exactly equal proportions of right and left sided breast cancers (49% and 51% respectively). The majority of tumours were ER+ve (see Table 1

**Table 1:**
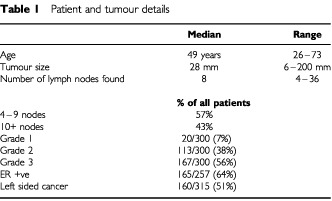
Patient and tumour details

for details).

### Treatment

The majority of women received all the planned chemotherapy (see Table 2

**Table 2:**
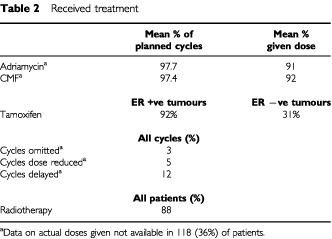
Received treatment

). Data on the details of treatment delivery are missing in 36% of patients, but of those for whom data are available, 43% of patients received every cycle exactly on time, and in 78% of patients (95% of cycles), treatment was at full dose. This resulted in the mean dose delivery for both adriamycin and CMF of over 90% (see Table 2). Even when including the patients with missing data, a minimum of 51% and 50% respectively of patients received 100% of the planned dose of adriamycin and CMF dose. For the vast majority of patients this was achieved without the use of G-CSF, which was not used in at least 224 patients, with only eight patients known to have received it.

Radiotherapy was given to 88% of patients, although a breakdown by the extent of loco-regional surgery is not available. Tamoxifen was recorded as given to over 90% of patients with ER +ve tumours, and almost one-third (31%) of women with ER −ve tumours.

### Outcome

**Figure 1:**
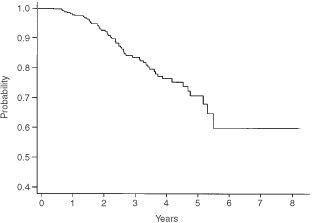
Overall survival for all patients with 4+ nodes.

**Figure 2:**
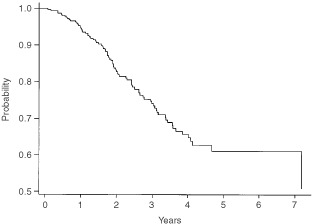
Disease-free survival for all patients with 4+ nodes.

**Figure 3:**
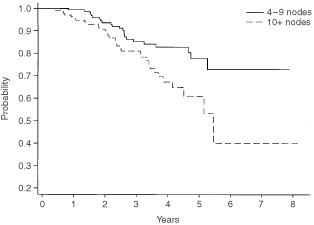
Overall survival by number of involved nodes.

The overall survival for the whole cohort is shown in Figure 1

, where it can be seen that the actuarial 5-year survival is 70%. Figure 2

shows the disease-free survival, which is 61% at 5 years. These data are shown separately for those women with 4–9 nodes and 10+ nodes respectively in Figures 3

and 4

**Figure 4:**
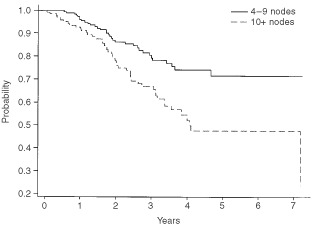
Disease-free survival by number of involved nodes.

. It should be noted that the 5-year disease-free and overall survival has not yet been reached for those women with between four and nine involved axillary nodes, whereas the figures are respectively 4 and 5.47 years for those women with at least 10 involved axillary nodes.

For the 33 (10%) of women who did not complete all 12 cycles of chemotherapy, there was no evidence of a poorer survival (data not shown), apart from the eight for whom treatment was stopped because of a recurrence of breast cancer during their adjuvant therapy.

Furthermore there is no evidence of a failure to deliver this therapy to older women, nor any suggestion that their outcome is poorer as compared with younger women in the audit. It is clear from Table 3

**Table 3:**
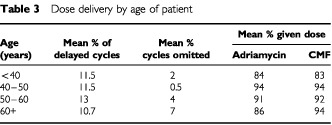
Dose delivery by age of patient

that although there was a trend towards omitting more cycles in the older patients, this did not translate into an overall lower mean dose delivery or a poorer survival. The median survival has not been reached in any age group (under 40, 40–50, 50–60 or over 60 years ) with no evidence of differential survival by age (*P*>0.4 for any comparison) (data not shown).

Table 4

**Table 4:**
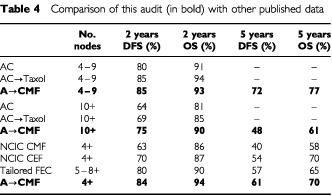
Comparison of this audit (in bold) with other published data

shows these figures and the available outcome data for patients with four or more involved nodes in both the NCIC MA5 study and the CALGB 9344. It can be seen that patients in this audit of routine practice do not appear to have a survival any poorer than the women in either the CEF or AC-Taxol arms of these two North American randomised trials.

## DISCUSSION

This audit was performed to determine the efficacy of the block sequential adriamycin-CMF regimen in non-trial patients. It is clearly an effective regimen, and although the patients are by definition a more heterogeneous group than those entered into clinical trials, the outcome data observed compare very favourably with the 5-year data seen in the original report of this regimen from the Institute Tumori di Milano. Indeed the fact that the outcomes appear even better than in that original report, as well as the 10-year data, may be due to the use of tamoxifen in almost all women with ER positive tumours in this series of patients, whereas it was not mandated in the original study. Another difference between these data and the original report is the outcome for the older woman: in the original Milan experience it is clear that the post-menopausal women fared worse, whereas in this audit there is no evidence of a poorer outcome. However, imbalances in other prognostic factors could have contributed to these differences in outcome from that earlier report. Whatever the reason, the most important observation is that when translated to routine practice, there has been no loss of efficacy for this regimen. This is an unusual occurrence in oncology, with the much more common pattern being less efficacy seen outside of a pivotal clinical trial.

The audit also demonstrated that this regimen is deliverable to the majority of patients despite involving 36 weeks' chemotherapy. Assessing toxicity is difficult in this type of retrospective audit, but there were no reports of toxic deaths or major morbidity. Furthermore, the small number of patients who failed to complete all the planned therapy, and the high mean dose delivered are further testimony to the applicability of this treatment in routine practice.

Having thus demonstrated that this regimen can be delivered outside clinical trials without any apparent loss of efficacy, its place must now be determined relative to those regimens that have more recently been reported to be superior to previous standards of care. A criticism of the adriamycin-CMF regimen is that it has never been compared with classical CMF. This omission will in large part be addressed by two parallel UK randomised trials which have compared CMF with a regimen consisting of four cycles of single agent epirubicin followed by four of CMF. The results of these two trials are awaited, although it has to be admitted that in these two studies, the CMF was given for four rather than eight cycles, and adriamycin was substituted by the less cardiotoxic agent epirubicin. Indirect comparisons can be made with data from other studies, although such analyses must be interpreted with caution and are in effect hypothesis generating rather than confirmatory.

Firstly, the NCIC MA5 trial in which methotrexate was substituted by epirubicin in six cycles of classical CMF convincingly showed that the use of anthracyclines can further improve the outlook for women with node positive breast cancer. Data on the 4+ node group are not available from the original publication, but have been made kindly available by the company that supported the study (Pharmacia). It can be seen in Table 4 that not only is the adriamycin-CMF regimen clearly superior to the classical CMF control arm, but could even be better than the CEF arm! The second data set with which a comparison can be made is the CALGB 9344 trial, in which a double randomisation occurred, between three different doses of adriamycin within four cycles of AC, and then to either four cycles of taxol or no further chemotherapy. The data have been presented on several occasions, but as yet no peer-reviewed publication has appeared. Therefore the data on the 4–9 and 10+ node subgroups are only available from a pharmaceutical company publication. It can again be seen in Table 4 that the outcome of patients in this audit is superior to the control arm of AC, and apparently equivalent to AC followed by taxol. Given that AC has been shown to be equivalent to classical CMF in both node negative and node positive patients, this further substantiates the view that adriamycin-CMF is superior to four cycles of AC or six cycles of CMF, and appears to be at least as effective as either AC-Taxol or days 1 and 8 CEF.

This audit has clearly demonstrated the efficacy outwith clinical trials of the sequential adriamycin-CMF regimen for women with high risk early breast cancer. Furthermore, indirect comparisons with the outcome data for two other regimens when given to this same high risk group show no evidence that they are superior. The results of the Anglo-Celtic I trial which compared the sequential adriamycin-CMF regimen with a myeloablative treatment are awaited, but unless that trial is positive (which would somewhat go against the trend of other recently reported high dose trials), then the adriamycin-CMF regimen would appear to be deliverable in routine practice, with as good an outcome as any other regimen.

Two other studies of adjuvant chemotherapy in high risk women have been published recently. One, the small Dutch high dose trial, did not define risk by the number of pathological nodes involved, and so comparisons with the current data set are difficult (Rodenhuis *et al*, 1998). The other, the interesting Swedish study of ‘tailored’ FEC against high dose chemotherapy, defined risk by the anticipated 5 year outcome with standard therapy (Bergh *et al*, 2000). This translated into including women without low-grade tumours, and either eight or more nodes when the tumour was ER positive or five or more nodes when it was ER negative. The outcome data for this study have been estimated from the published survival curves, and given that the patients in the Swedish study were at a slightly higher risk than the 4+ criterion for this study, the outcomes are again reasonably comparable.

We conclude that appropriately sequenced anthracycline based polychemotherapy is a standard of care for high risk breast cancer that, from indirect comparisons, has not been improved upon by the addition of taxanes or by escalation of dose. It remains to be seen whether the sequenced anthracycline regimen can be bettered with a different taxane, such as docetaxol, or by ‘tailoring’ the regimen to either the patients' tolerability or an approach using markers of drug sensitivity which have been derived from an individual's tumour biology.
